# Fetal volvulus: is emergency surgery the only option?—a single-center case series and management considerations

**DOI:** 10.1007/s00404-025-08256-4

**Published:** 2026-04-08

**Authors:** Maximilian Holweg, Justus Lieber, Karl Oliver Kagan, Cornelia Wiechers, Bianca Haase, Marla Delbrück, Markus Dietzel, Tobias Jhala, Christoph Slavetinsky, Andreas Schmidt, Jörg Fuchs

**Affiliations:** 1 Department of Pediatric Surgery and Pediatric Urology, University Children’s Hospital Tübingen, Hoppe-Seyler-Strasse 3, 72076 Tübingen, Germany; 2 Department of Obstetrics and Gynecology, University Hospital Tübingen, Tübingen, Germany; 3 Department of Neonatology and Neonatal Intensive Care Unit, University Children’s Hospital Tübingen, Tübingen, Germany

**Keywords:** Fetal volvulus, Intrauterine volvulus, Emergency surgery, Emergency cesarean section, Cystic fibrosis

## Abstract

**Background:**

Fetal intestinal volvulus is a rare intrauterine condition associated with potentially severe neonatal morbidity. Owing to its rarity, diagnostic features and management strategies remain heterogeneous and are largely based on case reports and small case series.

**Methods:**

We performed a retrospective single-center analysis of all patients diagnosed with intestinal volvulus between 2005 and 2022, comparing antenatally diagnosed fetal volvulus and postnatal presentation, diagnostic findings, management approaches, and outcomes. Given the rarity of fetal volvulus, the analysis was descriptive in nature.

**Results:**

45 patients with confirmed volvulus were analyzed, including seven fetal and 38 postnatal volvulus cases. Five fetal cases required postnatal surgical intervention, whereas two extremely preterm fetuses were managed conservatively with close prenatal surveillance and showed spontaneous regression of sonographic findings. Prenatal diagnosis relied mainly on ultrasound features, such as bowel distension and whirlpool sign. Outcomes in both groups were strongly influenced by gestational age and prematurity-related morbidity.

**Conclusion:**

Fetal volvulus presents with heterogeneous clinical courses and requires individualized, multidisciplinary decision-making. Conservative observation may be considered in selected stable cases diagnosed at extreme prematurity, whereas at more advanced gestational ages, signs of fetal deterioration may prompt consideration of delivery and postnatal surgical management. Due to the limited cohort size, no standardized treatment recommendations can be derived.

## What does this study add to the clinical work

**Table:** 

This study highlights the heterogeneous clinical course of fetal volvulus and underscores the importance of individualized, gestational age-dependent decision-making in a multidisciplinary setting. In selected stable cases diagnosed at extreme prematurity, close prenatal surveillance may be considered; however, signs of fetal deterioration at any gestational age may necessitate timely delivery and postnatal surgical management.	

## Introduction

Postnatal volvulus is a rare, acute, and life-threatening condition often associated with significant morbidity and mortality [1–4]. Most cases of midgut volvulus occur within the first year of life [1, 4, 5] and are frequently linked to abnormalities in bowel rotation [3, 5–7]. Emergency surgery is the treatment of choice with the outcome largely determined by the extent of intestinal necrosis, the need for intestinal resection, and the duration of parenteral nutrition (PN) [1, 2, 8].

Fetal volvulus, or intrauterine volvulus, is an even rarer condition [9, 10], characterized by the absence of distinct clinical signs in both the mother and the unborn child. The identification of typical signs of volvulus through fetal abdominal ultrasound is crucial for early diagnosis and risk assessment [9–16]. Despite its rarity, a limited number of case reports and small series have highlighted this prenatal emergency [10, 17, 18]. However, there is no established consensus on the optimal management approach, which can range from conservative observation to urgent cesarean section (CS) followed by immediate laparotomy of the newborn [9, 10, 14–19].

This study aims to descriptively analyze patients with fetal and postnatal midgut volvulus treated at our institution, focusing on diagnostic findings, management strategies, and outcomes.

## Methods

### Patients and ethical considerations

All patients with fetal and postnatal midgut volvulus treated at our institution between 2005 and 2022 were retrospectively analyzed, with care provided through the close collaboration of the Prenatal Diagnostics Unit (DEGUM III) for complex intrauterine cases, the Neonatology Center, the Pediatric Surgery Center and the Center for Chronic Intestinal Failure and Intestinal Rehabilitation. Demographic data, maturity of the neonates, weight, clinical, radiological, and intraoperative findings, treatment, as well as outcomes were collected from hospital records. Diagnostic, treatment, and outcome of fetal and postnatal volvulus were compared. This study was approved by the local Ethical Committee (number 371/2022BO2).

Statistical analysis was conducted using GraphPad Prism version 10.1.1 (GraphPad Software, San Diego, CA). Group comparisons were performed using the unpaired t test or the Mann–Whitney *U* test, as appropriate, while categorical variables were analyzed using Fisher’s exact test. The level of significance was set to *p* < 0.05.

## Results

### All patients

Over the 17-year study period, 56 patients treated for midgut volvulus at our center were identified. Of these, 54 underwent surgical intervention. To ensure a homogeneous sample, eleven patients with a history of prior abdominal surgery were excluded from further analysis. The resulting study population consisted of 45 patients: 38 (84%) were diagnosed postnatally and underwent emergency surgery, while seven (16%) were diagnosed prenatally with fetal volvulus. Among these prenatal cases, five required surgery and two were managed conservatively without intervention.

### Patients’ characteristics

The characteristics of the patient cohort are summarized in Table 1. Among all cases, 84% of diagnoses were made within the first year of life, excluding the two conservatively managed fetal patients. All patients in the fetal volvulus group were female. Conservatively managed patients were delivered at term, whereas surgically managed patients were born preterm.

**Table 1:** Clinical characteristics of patients with fetal and postnatally diagnosed volvulus

	Fetal volvulus (n = 7)				Postnatal volvulus (n = 38)		p value
Management	Conservative		Surgical		Surgical		
N	2		5		38		
Sex	♂ = 0, ♀ = 2		♂ = 0, ♀ = 5		♂ = 22, ♀ = 16		
Median age at diagnosis [days]	-		0		22.5 [1–4146]		p < 0.0001 (***)
mean age at diagnosis [days]	-		0		320 ± 132 (σ = 813.7)		p = 0.388
preterm infant (n)	0		5		14		p = 0.0161 (*)
mean GA of preterm infant [completed weeks of gestation]	-		32.8 ± 0.9 (σ = 2.1) [31–35]		27.9 ± 1.2 (σ = 4.6) [23–36]		p = 0.0336 (*)
mean birth weight of preterm infant [g]	-		2,146 ± 223.6 (σ = 500.0) [1,600–2,800]		1,177 ± 262.0 (σ = 980.4) [370–3,270]		p = 0.0522
mean GA at diagnosis [completed weeks of gestation]	24.5 ± 0.5 (σ = 0.7) [24, 25]		32.8 ± 0.9 (σ = 2.1) [31–35]		-		p = 0.0031 (**)
mean GA at birth [completed weeks of gestation]	38 ± 0.0 (σ = 0.0) [38.0–38.0]		32.8 ± 0.9 (σ = 2.1) [31–35]		-		p = 0.0194 (*)
mean birth weight [g]	2,880 ± 180.0 (σ = 254.6) [2,700–3,060]		2,146 ± 223.6 (σ = 500.0) [1,600–2,800]		-		p = 0.1157
CF	0 (0%)		2 (40%)		1 (3%)		p = 0.0587 ^a^ p > 0.9999 ^b^ p = 0.0316 ^c^ (*)

*GA* gestational age. a: Comparing fetal volvulus group to postnatal volvulus group regarding CF. b: Comparing conservatively to surgically managed fetal volvulus group regarding CF. c: Comparing surgically managed fetal volvulus to postnatal volvulus group regarding CF

In the postnatal volvulus group, the most common clinical symptoms were bilious or non-bilious vomiting (61%), acute abdomen (55%), and circulatory insufficiency or instability (24%).

**Fig. 1:**
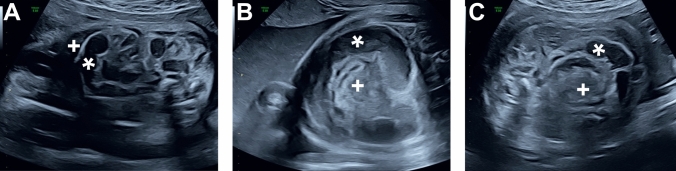
Prenatal ultrasound of a fetal volvulus. Female patient, 35 + 1 weeks of gestation. **A** Distended small bowel (*) and ascites (+). **B** Coffee bean/whirlpool sign (* and +) with distended small bowel (*). **C** Distended small bowel (*) and obstructed small bowel (+) due to suspected fetal volvulus

**Fig. 2:**
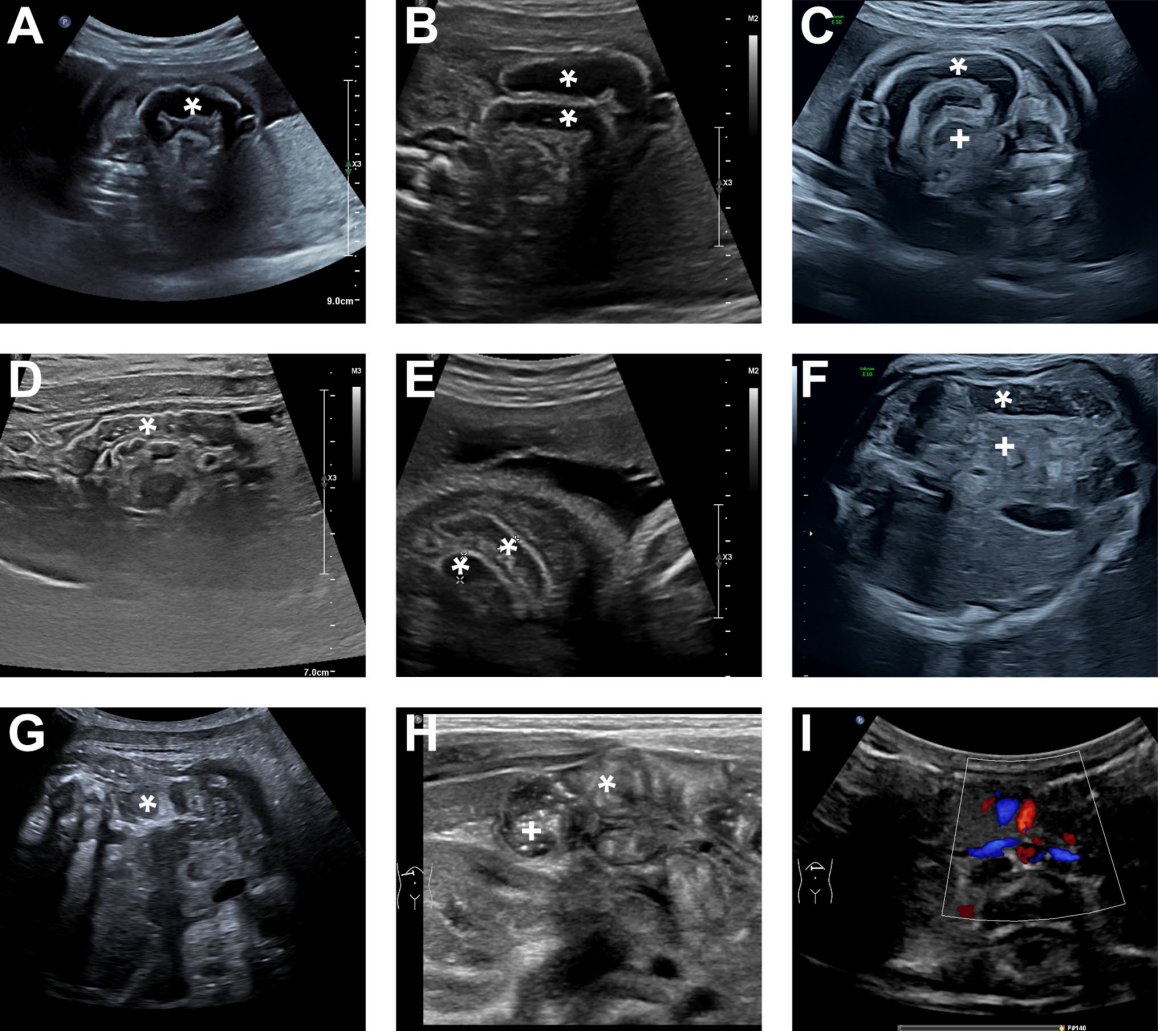
Fetal volvulus with typical prenatal ultrasound findings at 25 weeks of gestation without further symptoms. Due to extreme prematurity, conservative management was the rationale. Sonographic follow-up shows spontaneous detorsion of the volvulus and delivery was at term (38.3 weeks of gestation) without further problems. **A** Fetal ultrasound at a gestational age of 25 weeks. A dilatation (*) of small bowel loops up to 10 mm was noted in the lower abdomen, with a hyperechogenic bowel wall and detectable peristalsis. The bowel loops are arranged in a rounded, coiled manner (*), consistent with the appearance of a whirlpool sign, raising suspicion of a volvulus. **B** Fetal ultrasound at a gestational age of 25 weeks showing coffee bean sign (*) suspecting fetal volvulus. **C** Fetal ultrasound at a gestational age of 25 weeks showing coffee bean/whirlpool sign (* and +) suspecting fetal volvulus. **D** Fetal ultrasound at a gestational age of 28.6 weeks: The previously observed imaging findings suggestive of a volvulus have markedly regressed. Bowel dilatation was less pronounced (6 mm*), hyperechogenic bowel areas were only focally visible, and there was no evidence of free intra-abdominal fluid. **E** Fetal ultrasound at a gestational age of 31.6 weeks showing regression of bowel dilatation with normal bowel diameter. A short segment still showed areas with mildly increased wall echogenicity and discreet luminal dilatation. No signs of a clinically significant obstruction were observed. **F** and **G** Fetal ultrasound at a gestational age of 36.6 weeks showing normal bowel anatomy with normal bowel diameter (*) and normal mesentery (+). The bowel loops showed mildly increased peristaltic activity. **H** Postnatal ultrasound showing normal bowel anatomy with normal bowel diameter (*) and bowel filled with stool (+). **I** Postnatal ultrasound showing normal anatomy of the superior mesenteric vein and artery without sign of torsion

Prenatally, all patients with fetal volvulus exhibited volvulus-specific ultrasound findings summarized in Table 2, including bowel distension (100%) (Fig. 1, Fig. 2), and positive whirlpool or coffee bean sign (100%) (Fig. 1, Fig. 2). Additional nonspecific signs (Table 2) included pathological Doppler sonography with fetal centralization (60%), reduced fetal movements (40%), elevated middle cerebral artery (MCA) velocity (40%), abnormal cardiotocography (CTG) (40%), polyhydramnios (20%), ascites (40%) (Fig. 1), and suspected bowel necrosis or perforation (60%).

**Table 2:** Prenatal symptoms and ultrasound findings in fetal volvulus cases

	*n*
Symptoms	
Reduced fetal movement	2
Pathological CTG	2
Ultrasound findings	
Pathological prenatal Doppler sonography with fetal centralization	3
Polyhydramnios	1
Ascites	2
Bowel distension	7*
Whirlpool/coffee bean sign	7*
Intra-abdominal calcifications	0
Abdominal mass	0
Increased abdominal circumference	0
Elevated velocity in MCA	2
Meconium cysts/Pseudocysts	0
Suspected bowel necrosis/perforation	3
Pericardial and pleural effusions	0
Heart failure	0
Fetal death	0

Asterisks (*) denote features also present in conservatively managed fetal cases

Notably, the prevalence of cystic fibrosis (CF) in the surgical managed fetal volvulus group was 40%, representing a statistically significant difference compared to the postnatal volvulus group. CF was detected in all affected patients through routine newborn screening. An overview of all fetal volvulus cases, including gestational age (GA) at diagnosis, prenatal findings, management approach, and outcomes, is provided in Table 3.

**Table 3:** Summary of fetal volvulus cases (*n* = 7)

	GA at diagnosis (weeks)	Key prenatal ultrasound findings	Signs of fetal compromise	GA at delivery (weeks)	Management approach	Postnatal surgery	Outcome
1	24 + 6	Bowel distension, whirlpool sign	No	38 + 1	Conservative antenatal observation	No	Alive, normal feeding
2	25 + 0	Bowel distension, whirlpool sign	No	38 + 2	Conservative antenatal observation	No	Alive, normal feeding
3	31 + 1	Bowel distension, whirlpool sign, suspected perforation	No	31 + 1	Urgent CS	Yes	Alive, normal feeding
4	31 + 3	Bowel distension, whirlpool sign, ascites, suspected perforation polyhydramnios	Pathological CTG	31 + 3	Urgent CS	Yes	Alive, normal feeding
5	32 + 2	Bowel distension, whirlpool sign, abnormal Doppler	Pathological CTG, fetal centralization	32 + 2	Urgent CS	Yes	Alive, normal feeding
6	35 + 0	Bowel distension, whirlpool sign, ascites, suspected perforation abnormal Doppler	Reduced fetal movements, fetal centralization, elevated velocity MCA	35 + 0	Urgent CS	Yes	Alive, normal feeding
7	35 + 1	Bowel distension, whirlpool sign, abnormal Doppler	Reduced fetal movements, fetal centralization, elevated velocity MCA	35 + 1	Urgent CS	Yes	Alive, SBS, successful weaning of PN, normal feeding

*GA* gestational age, *CS* Cesarean section

Management decisions were made on an individual basis within a multidisciplinary setting and were not based on predefined treatment algorithms

### Surgery

Regarding the postnatal volvulus group, 23 of 38 patients showed malrotation, but none in the fetal volvulus group. In the postnatal volvulus group, additional findings included bowel necrosis in 12 out of 38 cases, necessitating bowel resection in these instances (Table 4). One patient presented with small bowel duplication, which required resection of the duplicated bowel, though no necrosis due to volvulus was observed. An intestinal stoma was created in ten patients (Table 4), with four of them requiring more than one stoma. Additionally, four patients needed the placement of an abdominal drain.

**Table 4:** Surgical and outcome parameters in the fetal and postnatal volvulus groups

	Fetal volvulus (*n* = 7)		Postnatal volvulus (*n* = 38)	*p *value (of surgical managed patients)
Management	Surgical	Conservative	Surgical	
*n*	5	2	38	
Malrotation	0 (0%)	0 (0%)	23 (61%)	*p* = 0.0161(*)
Primary bowel necrosis	3 (60%)	0	12 (32%)	*p* = 0.3236
Primary bowel resection	4 ^a^ (80%)	0 (0%)	13 ^b^ (34%)	*p* = 0.0707
Stoma	4 (80%)	0 (0%)	10 (26%)	*p* = 0.0322(*)
Primary SBS	0 (0%)	0 (0%)	2 (5%)	*p* > 0.9999
Secondary SBS	1 (20%)	0 (0%)	4 (11%)	*p* = 0.4786
Long-term PN	1 (20%)	0 (0%)	2 (5%)	*p* = 0.3164
Oral feeding	5 (100%)	2 (100%)	32 (84%)	*p* > 0.9999
Successful weaning of PN	1 (20%)	0 (0%)	1 (3%)	*p* = 0.2215
Mortality	0 (0%)	0 (0%)	5 ^c^ (13%)	*p* > 0.9999

a: One patient exhibited bowel perforation without necrosis, necessitating resection. b: One patient presented with an additional small intestinal duplication, which was surgically removed. c: All patients were born extremely preterm, with a mean gestational age of 25 weeks and a mean birth weight of 509 g

In the fetal volvulus group, bowel necrosis was observed in three patients. Resection of a portion of the small bowel was necessary in four cases, including one case of perforation without necrosis. Ileostomies were performed in four patients following primary bowel resection, and no abdominal drains were required.

### Conservative management

In two patients, fetal volvulus was diagnosed at extreme prematurity at 24.6 and 25.0 weeks of gestation, respectively (Fig. 2). Both patients were initially identified during routine prenatal check-ups by their community-based gynecologists—one due to fetal hydronephrosis and the other due to suspected intestinal atresia—and were subsequently referred to our center for further evaluation and management. Prenatal ultrasound in both cases revealed typical signs of fetal volvulus (Table 2), including dilated bowel loops and the characteristic whirlpool sign, with otherwise age-appropriate fetal growth and no additional symptoms. Given the extreme prematurity and the associated high risk of peri- and postnatal morbidity and mortality, a multidisciplinary team opted for a conservative, observational management approach with regular sonographic follow-up (Fig. 2). Regression of the sonographic findings was first observed 18 days after the initial diagnosis in one patient and 38 days after diagnosis in the other. Marked regression was documented after 25 and 77 days, respectively, and a normal ultrasound examination was achieved 24 and 77 days after diagnosis. At no point did either fetus exhibit additional symptoms. Both infants were delivered at term at 38 + 1 and 38 + 2 weeks of gestation, with birth weights of 2,700 and 3,060 g, respectively. Postnatally, both neonates showed good adaptation and excellent feeding tolerance. No surgical intervention was required, and both were discharged in good health six days after birth.

### Outcome

**Fig. 3:**
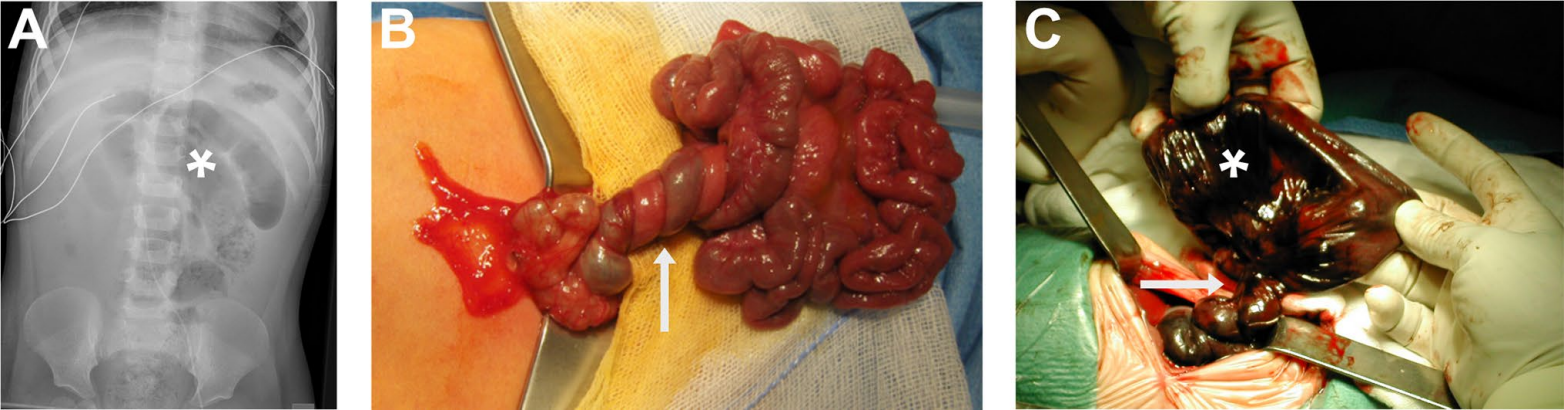
Configuration of volvulus in imaging and intraoperative situs. **A** X-Ray of the abdomen (posterior anterior projection) showing the coffee bean/whirlpool sign (*) as a sign of volvulus. **B** Intraoperative findings of midgut volvulus with multiple convolutions around the mesentery (arrow). The small bowel seems to be vital with sufficient perfusions. **C** Intraoperative finding of volvulus with multiple convolutions around the mesentery (arrow) resulting in necrotic small bowel (*)

All outcome parameters are presented in Table 4. In the postnatal volvulus group, five patients died during the study period. Four deaths were attributed to extensive bowel necrosis, while one occurred due to multi-organ and circulatory failure, leading to palliative care withdrawal. All deceased patients were extreme preterm infants with a mean GA of 25 weeks and a mean birth weight of 509 g [270–690]. These patients had developed short bowel syndrome (SBS) as a result of severe bowel necrosis (Fig. 3), and two exhibited intestinal malrotation.

Among the postnatal volvulus group, two patients presented with primary SBS due to extensive bowel necrosis after their initial surgery. Additionally, SBS developed in four other postnatal volvulus patients and one fetal volvulus patient as a consequence of subsequent surgeries. Notably, this cohort includes the four postnatal volvulus patients who succumbed to complications of bowel necrosis. All conservatively managed patients had a good outcome. Apart from the sonographic findings of a volvulus with a whirlpool sign and dilated loops, no additional symptoms, deterioration or instable vital signs were observed (Fig. 2).

A total of 24 out of 38 patients (63%) in the postnatal volvulus group and the five surgically managed patients (100%) in the fetal volvulus group required partial or total parenteral nutrition (PN) during hospitalization, but none of the conservative managed fetal volvulus patients. Following discharge, PN dependency persisted in two of the 24 postnatal volvulus patients and one of the seven fetal volvulus patients due to SBS. All three patients were enrolled in our intestinal rehabilitation program. One postnatal volvulus patient and the fetal volvulus patient were successfully weaned from PN, while the remaining patient, suffering from extreme SBS after postnatal volvulus, continues to depend on total PN.

Recurrent volvulus (re-volvulus) was observed in one patient in the postnatal group during follow-up, necessitating a repeat emergency surgery.

## Discussion

The main finding of this analysis is that fetal volvulus, when managed in a specialized center within a structured multidisciplinary setting, can be associated with favorable outcomes. The clinical course and management decisions, however, vary substantially depending on GA and the presence of fetal compromise.

In general, volvulus is associated with high morbidity and mortality [1–4]. Postnatally, there are symptoms that may indicate this rare disease and always necessitate emergency surgery if confirmed by imaging. In contrast, the diagnosis of a fetal volvulus is much more challenging, and the therapeutic procedure is not clearly defined. The aim of this study was to evaluate and compare different parameters of children with fetal and postnatal volvulus and to describe and compare clinical presentation, diagnostic features, management approaches, and outcomes of fetal and postnatal volvulus, and to discuss management considerations in the context of existing literature.

Diagnosis of fetal volvulus remains challenging due to its nonspecific presentation, the absence of distinct fetal symptoms, and the lack of definitive ultrasonographic or laboratory markers [1, 2]. In our cohort, prenatal suspicion of fetal volvulus primarily relied on sonographic findings of bowel distension and the characteristic whirlpool or coffee bean sign in all cases (Table 2, Fig. 1, Fig. 2), which served as the main diagnostic indicators.

Additional findings, while less specific, supported the urgency for intervention (Table 2). These included pathologic prenatal Doppler sonography findings, such as fetal centralization or elevated velocity in the MCA, reduced fetal movements, abnormal CTG, polyhydramnios, ascites, and sonographic signs, suggestive of bowel necrosis or perforation. These observations align with features previously reported in the literature [9–16, 18, 19] and led to evaluation of surgical management.

Other abdominal abnormalities described in the literature include abdominal masses and intra-abdominal calcifications, the latter often representing a consequence of bowel perforation [9, 10, 12, 13, 15, 16]. Reported complications include bowel necrosis, perforation, pericardial and pleural effusions, heart failure, and, in severe cases, fetal demise [9, 14, 15, 20].

If fetal volvulus is suspected, the management approach is primarily guided by the fetus's GA and clinical condition, as indicated by its vital parameters [9, 10, 12–16, 21]. Decision-making is conducted collaboratively within an interdisciplinary team in an obstetrics and pediatric center, including specialists in obstetrics, neonatal and pediatric intensive care, and pediatric surgery [9, 15]. While no standardized guidelines exist in the literature, management strategies range from close monitoring to urgent or emergency CS with immediate neonatal surgery [9, 10, 12–16, 18, 21].

All patients in our case series were preterm at diagnosis. The conservatively managed two patients with 24.5 completed weeks of gestation and the surgical managed five patients with a median age of 32.8 completed weeks of gestation. The youngest surgically managed patient was at a GA of 31 completed weeks of gestation. According to the classification of prematurity, the conservatively managed patients in our cohort are classified as extremely preterm (< 28 weeks of gestation) at the time of diagnosis, whereas the surgically managed patients belong to the categories of very preterm (28– < 32 weeks of gestation), moderately preterm (32– < 34 weeks of gestation), and late preterm (34– < 37 weeks of gestation). Patients diagnosed at a gestational age above 28 weeks are generally sufficiently mature to survive the neonatal period and may achieve favorable outcomes [22].

In our cohort, the two fetuses diagnosed before 28 weeks of gestation were managed conservatively. At this stage of extreme prematurity, the risks associated with delivery and postnatal surgical intervention, including high morbidity and mortality, may outweigh potential benefits [22]. In selected stable cases, conservative observation with close antenatal monitoring may therefore be considered [9, 10, 12–16, 18, 21]. Additionally, spontaneous detorsion of fetal volvulus has been described in the literature [18] and was observed in our cases, allowing continuation of pregnancy and delivery at term.

**Fig. 4:**
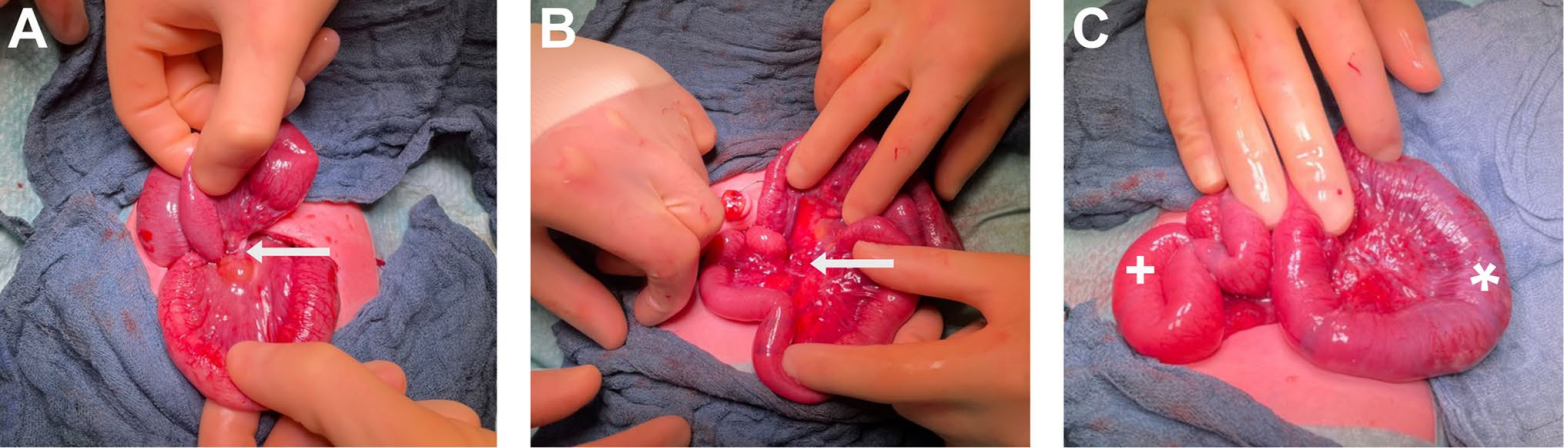
Intraoperative findings of a fetal volvulus. Female patient, 35 + 1 weeks of gestation, with distended and obstructed small bowel, ascites and coffee bean sign (Fig. 1) in the prenatal sonography after delivery via emergency cesarean section Emergency laparotomy of the newborn was performed. **A** Torquing of the bowel and mesentery. **B** Situs after derotation of the bowel and mesentery. **C** Situs after derotation with well perfused bowel (+) and livid bowel (*)

In contrast, in fetuses at more advanced gestational ages (beyond 28 weeks of gestation), management becomes increasingly dynamic and symptom-dependent. While observation may remain an option in stable cases with reassuring ultrasound findings, signs of fetal deterioration—such as pathological CTG, worsening Doppler parameters, or progressive sonographic abnormalities—may lead to consideration of urgent or emergency CS followed by immediate neonatal surgery (Fig. 4), in order to prevent severe complications such as bowel necrosis or perforation [9, 10, 12–16, 18, 21].

In two cases diagnosed at approximately 24 and 25 weeks of gestation, the fetuses were managed conservatively owing to extreme prematurity and the associated perinatal risks, despite the absence of additional clinical signs of fetal compromise. In contrast, in five cases, fetal distress—indicated by pathological CTG findings or deteriorating sonographic parameters—prompted urgent or emergency cesarean section, followed by immediate neonatal laparotomy. This management approach is consistent with strategies described in previous case reports and case series [9, 10, 12–16, 18, 21].

In our cohort, malrotation—a common finding in postnatal volvulus [1, 2, 5, 23, 24]—was notably absent in fetal volvulus cases, consistent with reports suggesting its lower impact in fetal volvulus [7, 11, 12, 16, 17, 19, 25–31]. A striking observation was the significantly higher prevalence of CF in the surgically managed fetal volvulus cases, with 40% of patients affected (*p* = 0.0316), but none in the group of conservatively managed fetal volvulus, leading to a CF incidence of 29% in overall fetal volvulus group. This aligns with literature suggesting a strong association between mutations in the Cystic Fibrosis Transmembrane Conductance Regulator (CFTR) gene, intrauterine intestinal obstruction, and fetal volvulus, with prevalence rates reported between 37.5 and 100% [9, 10, 20, 32]. CFTR mutations lead to highly viscous mucus, which may contribute to intestinal obstruction and serve as a potential pathomechanism for fetal volvulus [9, 10, 20, 32].

Despite these complexities, the overall outcomes for fetal volvulus in our cohort were favorable. All patients achieved oral feeding, and only one required long-term parenteral nutrition. These results are consistent with literature-reported survival rates of 80% and a SBS prevalence of 17% [19]. Regarding the three patients in our overall cohort requiring long-term PN—one in the fetal volvulus group and two in the postnatal group—two (one in each group) were successfully weaned after referral to our pediatric intestinal rehabilitation program, as recommended in literature [33–37].

These observations support conservative management as a possible option in selected cases of extreme prematurity (less than 28 weeks of gestation), particularly in the absence of fetal distress, in order to minimize the risks associated with surgical intervention in a highly immature fetus. However, they do not allow generalized treatment recommendations.

Overall, management of fetal volvulus requires careful, individualized decision-making based on gestational age, ultrasound findings, and fetal well-being within a multidisciplinary setting.

## Conclusion

Fetal volvulus is a rare condition with a heterogeneous clinical presentation and course. Management decisions should be individualized and based on gestational age, ultrasound findings, and fetal well-being within a multidisciplinary setting. Conservative observation may be considered in selected stable cases, particularly in extreme prematurity, whereas signs of fetal deterioration at more advanced gestational ages may prompt consideration of intervention. Early diagnosis and management by a specialized multidisciplinary team are crucial for favorable outcomes. Larger multicenter studies are needed before standardized management strategies can be defined.

## Data Availability

The datasets generated and/or analyzed during the current study are available in the article and its supplementary materials. Additional information is available from the corresponding author upon request.

## Abbreviations

CF: Cystic fibrosis
CFTR: Cystic fibrosis transmembrane conductance regulator
CS: Cesarean section
CTG: Cardiotocography
GA: Gestational age
MCA: Middle cerebral artery
PN: Parenteral nutrition
SBS: Short bowel syndrome
